# Tailoring interpersonal psychotherapy to psycho-oncology patients (TIPTOP): feasibility study protocol

**DOI:** 10.3389/fpsyt.2025.1531756

**Published:** 2025-04-25

**Authors:** Ebba Laing, Rita Acebo de Arriba, Elisabeth Schramm, Norbert Schäffeler, Stephan Zipfel, Andreas Stengel, Johanna Graf

**Affiliations:** ^1^ Department of Psychosomatic Medicine and Psychotherapy, Psycho-Oncology Division, University Hospital Tübingen, Tübingen, Germany; ^2^ Comprehensive Cancer Center Tübingen-Stuttgart, University Hospital Tübingen, Tübingen, Germany; ^3^ Department of Psychiatry and Psychotherapy, Medical Center– University of Freiburg, Faculty of Medicine, University of Freiburg, Freiburg, Germany; ^4^ German Center for Mental Health (DZPG), Tübingen, Germany; ^5^ Clinic für Psychosomatic Medicine and Psychotherapy, Klinikum Stuttgart, Stuttgart, Germany; ^6^ Stuttgart Cancer Center (SCC), Klinikum Stuttgart, Stuttgart, Germany

**Keywords:** interpersonal psychotherapy (IPT), group psychotheraphy, feasibility studies, psycho-oncology, mood disorder, psychological distress

## Abstract

**Objective:**

Patients with cancer suffering from comorbid symptoms of depression need a psychotherapeutic treatment that is specifically tailored to the exceptional context of acute or chronic cancer treatment. The conceptualization of depression in Interpersonal Psychotherapy (IPT) is a promising framework for symptoms of depression in patients with cancer, because it focuses on coping with stressful life events, managing change and accessing social support.

**Method:**

The intervention was developed based on standard practice of group IPT for depression and adapted to the oncology setting by an expert panel. The IPT intervention comprises 10 weekly sessions of 60 minutes each. It is structured into modules incorporating IPT core themes: role transitions, grief, connection and interpersonal conflict. This feasibility study with an integrated qualitative study seeks to ascertain feasibility and acceptability of a larger trial of modified group IPT for adult patients with cancer within the context of a university clinics’ outpatient treatment. In addition, the study will enable first evidence of the intervention’s efficacy in reducing symptoms of depression and anxiety, and changes in interpersonal factors such as loneliness, thwarted sense of belonging, perceived burdensomeness, and perceived social support. Post-intervention semi-structured interviews will be recorded for qualitative content analysis.

**Conclusion:**

Our findings will suggest whether investigating group IPT as proposed is an acceptable, feasible and safe approach to ameliorate symptoms of depression in patients with cancer. The study is innovative in that it provides a new treatment to patients of different cancer types and treatment stages, creating a setting that is naturalistic and realistic in the context of psycho-oncology services.

## Introduction

The effects of cancer diagnosis and corresponding cancer treatment on the patients’ mental health have been well documented in the past decades (1–3). Across different populations and treatment settings one in two patients with cancer report significant distress (3), and a subset of 29% fulfill DSM-5 diagnostic criteria of mood disorders (2). Depression in patients with cancer can lead to poorer quality of life and that itself warrants psychological treatment. However, it also has effects beyond the mental health of the patients in that they are more likely to struggle with treatment decisions and may require more intensive attention from the medical team (4, 5). In addition, patients with cancer affected by depression are less likely to comply with treatment regimens (6), and untreated comorbid depression and distress have been linked to shorter survival in meta-analyses (7). Beyond that, the patients’ mental health affects their interaction with caregivers and thereby the caregivers’ well-being and caregiver burden. Therefore, comorbid symptoms of depression in patients with cancer warrant timely, specific, and intensive psychotherapeutic treatment (8). Standard psychotherapeutic treatment for depression, however, is not adapted to the specific context of symptoms of depression embedded in an oncological illness. There is a range of important characteristics that a specific psychotherapy for symptoms of depression associated with cancer should include:

- Access to the psychotherapy should be low threshold, in order to facilitate enrollment for patients in crisis.
- It should keep the additional treatment burden on patients as low as possible and yield fast and palpable benefits to keep patients motivated and adhering.
- It should be feasible for patients and providers in diverse treatment stages and treatment settings of psycho-oncology care facilities.
- Conceptually, the psychotherapy should reflect the cancer-related etiology of symptoms of depression by addressing stressors specific to cancer patients’ experiences. Such stressors are, for example, changes in everyday life and relationships, declines in quality of life, struggles with treatment demands, communication with the medical treatment team, acute confrontation with mortality, isolation and lack of meaningful connection, and grief for a lost healthy self.
- The psycho-educative framework of symptoms of depression ought to be comprehensible and acceptable to patients with cancer and to their caregivers.
- The psychotherapy should be empirically tested for effectiveness and benefit for the patients.

There is one framework for the psychotherapeutic treatment of depression that appears to be an ideal fit with the needs and requirements of this patient group: it is the conceptualization of depression in Interpersonal Psychotherapy (IPT).

IPT was developed by Klerman et al. (9) to provide treatment for episodic unipolar depression. Drawing on the interpersonal theory of psychiatry by Sullivan (10), and on J. Bowlby’s attachment theory (11) as expanded on by Rutter (12), they conceptualized the development of symptoms of depression as inextricably intertwined with interpersonal factors such as social roles, interpersonal relations, and life events. IPT sees interpersonal difficulties as both antecedents and consequences of clinical depression, with the two continuously reinforcing each other. The interpersonal approach to treating depression is to ameliorate the patients’ interpersonal difficulties, centered in four focal areas: role transitions, conflict, isolation and loneliness), and grief besides building up social support. The patient is educated about depression and assigned the ‘sick role’ (13). This clarifies that the patient is temporarily in need of help and takes away the burden of responsibility, guilt or shame for the symptoms.

The effectivity of IPT in treating depression has been thoroughly evaluated (14) and IPT is among the recommended treatments for depression in international practice guidelines (15). IPT has also proved effective in treating anxiety disorders (16). Globally, IPT has been applied to highly diverse patient populations in a variety therapeutic settings (17).

The interpersonal conceptualization of symptoms of depression is plausible and acceptable to patients with cancer and their relatives or caregivers, because it offers them not yet another incomprehensible diagnosis but a rationale with a comprehensible, strategic treatment plan. By taking on the ‘sick-role’, patients learn to understand their depressive symptoms not as a personal failure, but as a reaction to the marked changes in important interpersonal areas brought on by the cancer diagnosis and treatment.

**Figure 1:**
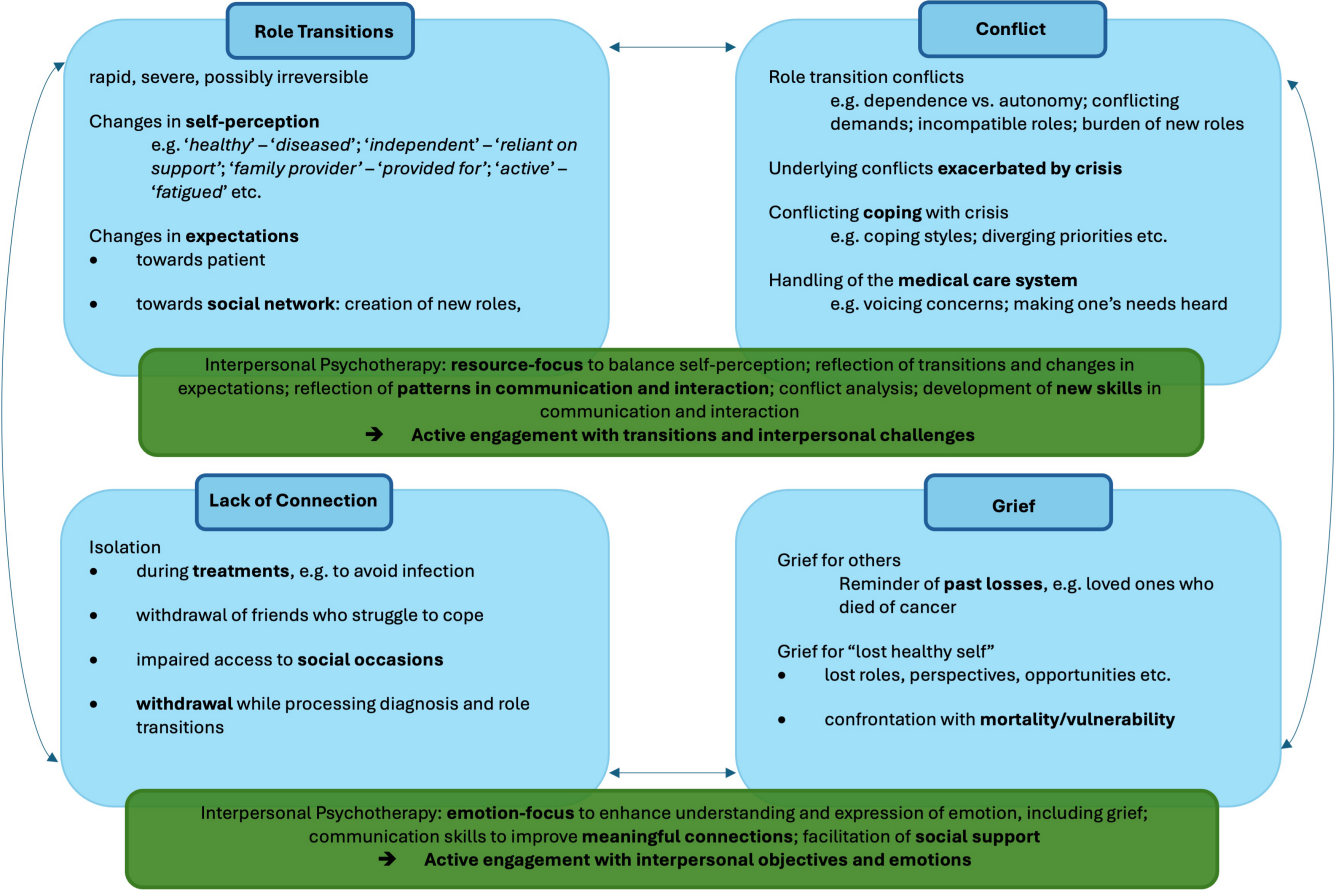
Application of Interpersonal Psychotherapy Focal Areas in Cancer.

The focal areas of IPT (role transition, interpersonal disputes, isolation, grief) can also be applied to the context of oncology with significant benefit, as is shown in (**Figure 1**). As in the Interpersonal and Social Rhythm Therapy, an adaptation of IPT for bipolar disorder (18), the focal area of “grief” targets expression of grief over the “lost healthy self”.

IPT has been applied successfully to depression in other populations, such as HIV-seropositive patients (19), patients with heart disease (20), patients in rehabilitation after a stroke (21) and elderly patients (22). We assume that these patient groups share important characteristics with patients with cancer. They, too, may be faced with role transitions concerning loss of activity, strength, autonomy, and perceived immortality. Moreover, they may also be forced to navigate novel interpersonal challenges, and may face similar conflicts caused by the role transitions brought on by their health conditions. They may experience loneliness and isolation, feeling stigmatized and disconnected from their peer group. They, too, may be reminded of past losses of loved ones to similar ailments. The applicability of IPT in these conditions speaks to the conceptual applicability of IPT for patients with cancer.

A current systematic review of randomized controlled trials of IPT applications in oncology (23) shows a paucity of research on the subject. Six studies focused on remote Interpersonal Counselling (IPC), a shorter, more accessible form of IPT developed for primary health care settings (24–30). Results regarding the efficacy of remote IPC in oncology settings were inconclusive (23). Two studies investigated individual IPT in oncology settings. IPT proved to be superior to treatment as usual in reducing symptoms of depression in patients with cancer both these trials (31, 32).

A group psychotherapy concept appears to yield added benefits in the context of psycho-oncology, considering the alienating and isolating experiences of patients with cancer. Sharing with a group of similar experience can offer patients relief by normalizing psychological distress and stressors. From the very first session on, the group can offer patients a sense of connection, acting as a first intervention towards decreasing loneliness. Group members can also provide useful feedback when tackling interpersonal skills and conflicts. Thus, the group will aid in decreasing the psychological burden and in increasing motivation to keep participating.

The effectiveness of group IPT has been assessed in different populations, and for a variety of disorders, such as depression, binge eating disorder, and posttraumatic stress disorder (33), but so far not for depression in patients with cancer (23). Therefore, the aim of this study is to develop an IPT group program tailored specifically to the needs of patients with cancer and conduct a preliminary evaluation of the program. An effective, accessible treatment for symptoms of depression in patients with cancer will improve health care for patients with cancer not only by improving their quality of life and alleviating mood disturbance, but also by enabling them to more thoroughly adhere to cancer treatments.

## Objectives and research questions

The object of this study is to assess the feasibility and acceptability of modified group IPT for patients with cancer in outpatient settings in a longitudinal study design, and identify barriers and facilitators to the implementation of the intervention. The study also aims to preliminarily ascertain effects of the treatment to alleviate depression in patients with cancer and to reduce further facets of psychological burden (anxiety, cancer-related psychological distress, loneliness, perceived burdensomeness, thwarted belongingness) and improve perceived social support. All data collected will be used to inform the decision on progression to a full trial.

## Methods and analysis

### Study design

The study is a feasibility study, more specifically an internal non-randomized pilot study according to the framework proposed by Eldridge et al. (34), with an integrated qualitative study. It is designed to investigate the feasibility and preliminary efficacy of adapted group IPT for cancer patients in outpatient settings and therefore corresponds to steps one (feasibility) and two (efficacy) of the Translational Research Framework (35), guiding implementation research in health services. The obtained data will be used to inform a broader trial of the intervention.

**Figure 2:**
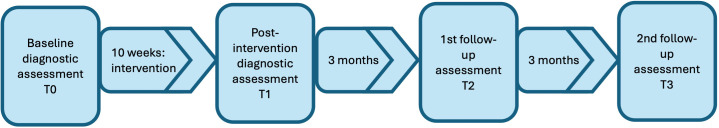
Trial Flow.

The trial flow is shown in (**Figure 2**). Informed consent will be obtained before participation. Participants will then complete the assessments online using the REDCap survey tool (36, 37). For an overview of diagnostic instruments, see (**Table 1**). After participating in the 10-week modified group IPT intervention, they will complete the same assessment again at T1. The post-intervention interview will be audio-recorded for analysis. Assessments will be repeated at follow-up T2 three months after completion of the intervention. The intervention is considered completed when participants have attended 8 out of the 10 sessions.

**Table 1:** Assessment schedule.

Measures			Baseline (T0)	Post-treatment 10 weeks after baseline (T1)	3- months follow-up (T2)	6-months follow-up (T2)
Sociodemographic and medical data			x	x (shortened^1^)	x (shortened^1^)	x (shortened^1^)
Primary Outcome	Symptoms of Depression	PHQ-9	x	x	x	x
Secondary Outcomes	Symptoms of Anxiety	GAD-7	x	x	x	x
	Psychological Distress	DT analogue scale and problem areas	x	x	x	x
Tertiary Outcomes (Mechanisms)	Loneliness	R-UCLA	x	x	x	x
	Perceived Social Support	BS6	x	x	x	x
	Interpersonal Needs	INQ	x	x	x	x
interpersonal stressors		Self-generated items	x	x	x	x
Quality of Life		EQ-5D-3L	x	x	x	x
Need for psychosocial support		HSI	x	x	x	x
Acceptability and Feasibility		Self-generated items		x		

DT, Distress Thermometer; HSI, Hornheider Screening Instrument; GAD-7, Generalized Anxiety Disorder Questionnaire; PHQ-9, Patient Health Questionnaire; R-UCLA, Revised UCLA Loneliness Scale; BS6, Brief Social Support Scale; INQ, Interpersonal Needs Questionnaire

^1^ items omitted at follow-up: age, gender, past psychiatric disorder, cancer entity, date of first cancer diagnosis

Results will be published according to CONSORT reporting guidelines (CONSORT 2010 statement: extension to randomised pilot and feasibility trials, 38), excluding the randomization section.

### Participant eligibility

Participants will be included provided they are 18 or older, give written, informed consent, have a diagnosis of cancer and are willing and able to participate in the online assessments and 10 weekly sessions of group IPT in person. A further inclusion criterion is self-reported symptoms of depression (rating of 3 or higher in PHQ-2). Since there are no exclusion criteria regarding time since cancer diagnosis, self-reported cancer-related distress (DT≥ 4) is used as an inclusion criterion as well, to ensure participants consider their cancer diagnosis relevant to the symptoms they are experiencing. Participants will be excluded if they report comorbid psychiatric disorders requiring more intensive treatment (acute psychotic symptoms, acute manic episode, acute suicidality, acute substance use disorder excluding nicotine) within the past four weeks, and if they are currently in regular treatment with a psychotherapist. Patients receiving antidepressant medication will be included if they agree to stay on a stable dose and medication throughout their participation in the study and agree to report changes in medication. There are no exclusion criteria regarding type of cancer or cancer treatment stage.

### Participant recruitment

Participants will be recruited from the pool of patients receiving treatment in oncological facilities of the University Hospital Tübingen (UKT), and the Comprehensive Cancer Center Tübingen-Stuttgart including the psychosocial counselling facilities (Krebsberatungsstelle Tübingen) using flyers, posters, social media outlets and information provided by nurses, doctors, and psychologists. Patients in in- and outpatient oncological care at the UKT complete an electronic psycho-oncological screening (ePOS) as part of routine cancer patient care and are approached by the psycho-oncology services if indicated (see 39, 40). If they indicate symptoms of depression (rating of 3 or higher on PHQ-9), psychological distress (rating of 4 or higher on DT analogue scale) on the ePOS, their psycho-oncologist will ask if they are interested in participating in the study. Participants who indicate interpersonal distress (i.e. “no” for “Do you have someone to talk to about your worries”, “yes” for “familial problems”) will also be informed about the program and will be included in the pilot study if they meet the inclusion criteria. Information about the treatment program and ways of referral will also be sent out to practitioners who might treat cancer patients (general practitioners, oncologists, gynecologists, urologists, neurologists, surgeries, etc.) in the area around the clinic. Questions regarding the study will be answered via email, phone or in person. Those seeking participation will be screened for inclusion and exclusion criteria. Participants will not receive compensation for participating in the study. Recruitment will end when 40 patients have completed assessments. For the qualitative study, recruitment will end when saturation is reached as described below.

### Intervention

Group IPT addresses current findings on comorbid mental health conditions in cancer patients. It aims to enable patients to reduce symptoms of depression by reflecting and adapting the way they handle interpersonal role transitions and stressors. It is based on evidence-based methods of IPT and has been adapted to the challenges patients with cancer encounter by an expert panel for psycho-oncology care within the University of Tübingen. Previous research showed the efficacy of individual IPT for depressed patients with breast cancer, patients with HIV, and elderly patients, which share important characteristics with patients with cancer (23).

By addressing directly the difficult role changes patients with cancer face and by reflecting the role of acceptance and personal values in that transition, group IPT adapted for patients with cancer can empower patients to proactively engage with the novel situation and foster a sense of participation in the transition. By addressing how that role change affects patients interactions with their support system, group IPT adapted for patients with cancer can empower patients to (re-)gain access to social support. The group context provides patients with a group of reference with which to share experiences. Emotion-focused elements provide them with ways to communicate feelings and needs directly and effectively. In using patients’ personal conflicts and practicing different ways of engagement, group IPT adapted for cancer patients increases patients’ repertoire of interpersonal behaviors, enabling them to interact more flexibly within the changing and novel demands of their new roles. Thus, group IPT adapted for cancer patients can reduce depressive symptoms by addressing interpersonal stressors such as helplessness, loneliness, and lack of range in communication and interaction skills.

#### Intervention structure

The IPT intervention encompasses 10 weekly sessions of 60 minutes each. It is structured into modules incorporating IPT core themes: psychoeducation, role transitions (incorporating grief for the “lost healthy self”), and connection/interpersonal conflict. (**Table 2**) shows sessions, session content, therapeutic goals and patient homework for each module. Pre- and post-group treatment sessions will be held as described in (**Table 3**) (see 41). In pre-group treatment sessions, participants will set individual goals that fit within the IPT core themes and will be encouraged to work on them in group sessions by applying the session content to their specific examples (e.g. in role-play sessions). Post-group sessions will include a semi-structured interview encompassing questions on goal attainment, interpersonal experiences (42), outlook and change maintenance and questions regarding barriers and facilitators to participation and unintended effects of the intervention. Groups will consist of at least three and at most ten participants at a time and will remain in the same constellation across all ten group sessions.

**Table 2:** Intervention modules description.

Session no.	IPT Problem Area	Content	Goals	Homework assignment
1	Psycho-education: cancer and mental health	• vulnerability-stress model • psychiatric comorbidities of cancer and “sick role” • resilience factors retained throughout cancer treatment • premises of IPT and application to struggles of patients with cancer	• Foster understanding of mental health issues in connection with cancer and treatment options • normalize distress/depression as a reaction to cancer diagnosis • empowerment • foster group coherence through shared experience of cancer diagnosis	• reflect strongest symptom of depression, apply coping/resilience as discussed
2	Role Transition: Introduction	• common role transitions for patients with cancer • transfer to individual role transitions • introduction to mindfulness exercises: 3-minute breathing space	• identify current changes and constants • reframe current changes as role transitions in wake of cancer diagnosis • normalize struggles adapting to change • foster group coherence by identifying shared struggles • foster reflection of individual, implicit connotations of new role	• reflect personal role transitions in wake of cancer diagnosis regarding three aspects: “what is lost?”, “what remains the same?”, “what is gained?”
3	Role Transition: emotion focus	• psycho-education: emotions • emotions commonly reported by cancer patients - fear/anxiety - shame - guilt - sadness - grief - hope	foster deliberate reflection of emotions • enhance emotional vocabulary and expression → prepare new interpersonal behaviors • reduce stigma of talking about uncomfortable emotions • encourage hope in spite of cancer diagnosis	deliberately observe emotional states and experiences throughout the week, take notes on coping strategies
4	Role Transition: Acceptance	• strategies for coping with challenging emotions commonly associated with cancer diagnosis/treatment • accepting uncomfortable emotions: living-with-your-monster metaphors	• educate about adaptive and maladaptive coping strategies • foster tolerance for uncomfortable emotions • support transition processes • foster serenity towards self and others	• try out new coping strategies, observe changes
5	Role Transition: Values	• psycho-education on values and mental health in context of personal health crisis • development of individual “compass of values” • develop individual behaviors to align new role with values	• foster reflection of and commitment to personal and interpersonal values • reframe current crisis as an opportunity for realignment with values and commitment to new values • create opportunities for post-traumatic growth from current crisis	• try one new value-based behavior
6	Role Transition: resources and isolation	• exploration and manifestation of personal resources (energy providers, energy takers) throughout cancer treatment • education on attention biases and connection to mood and fear of recurrence of cancer • introduction to gratitude exercises and self-compassion • lovingkindness meditation with application to cancer-specific struggles	• foster balanced view of lost and new roles • activate preexisting resources lost in cancer treatment • educate on possible new resources (gratitude journal, meditation) • foster sense of connection beyond oneself in current crisis	• implement one resource into everyday life • gratitude journal (continuously throughout the therapeutic process)
7	Connection	• education on communication • introduction to Kiesler Circle • transfer to individual and cancer specific interactions and difficulties • role-play and analysis: asking a doctor to repeat explanation of laboratory results	• foster self-efficacy in interaction • expand repertoire and flexibility of interpersonal behaviors esp. regarding medical context • strengthen dyadic coping	• use Kiesler Circle as framework for observation of interaction throughout the week
8	Connection	• Education on interpersonal relationship barriers and enablers • Practicing change of perspective • Transfer to Individual interactions: role-play and analysis with typical cancer-related situations	• expand repertoire and flexibility of interpersonal behaviors • apply theory to practice • strengthen dyadic coping	• try new behaviors • short analysis of a (conflict) interaction
9-10	Interpersonal Conflict	• education on conflict stages • practicing change of perspective • transfer to personal interactions: role-play and analysis with patients’ conflicts (iterating, chaining, shaping) • reflect changes in relationship connected to changes in interpersonal behavior	• foster deliberate, goal oriented reflection of conflict • expand repertoire of interpersonal behaviors • apply theory to practice • strengthen dyadic coping • strengthen/expand social network • evaluate and modify closeness circle	• try new behaviors • short situation-analysis of a cancer-related (conflict) interaction

**Table 3:** Intervention schedule.

Modality	Goals	Duration
Individual Pre-Group	Determine patients’ personal treatment goals, explore focal areas, explore patients’ social network (closeness circle), inform about IPT group therapy rationale; study information and informed consent	60 min
Group (10 sessions)	See (**Table 2**)	60 min each
Individual Post-Group	Determine goal attainment, ensure maintenance of changes, feedback and remaining questions	60 min

The group will be led by a licensed psychologist-psychotherapist and a psychologist. They will both complete a three-day training course in IPT and will be supervised monthly by an external experience IPT supervisor (E.S.).

#### Session structure

Every session begins with a short check-in (“How are you feeling coming in today? Are there things you would like to add regarding the previous session?”). After that, the previous session’s homework is discussed before opening the new session’s topic. Every session is concluded with a five-minute space for the participants to note down their personal take-home message(s), and a short check-in (“How are you feeling going home now?”, “Would you like to share a take-home message?”). After the short introduction to mindfulness practices in session two, all sessions are concluded with a joint mindfulness exercise (Three-minute breathing space).

To reflect adequately the context of outpatient psycho-oncology treatment, the intervention is deliberately aimed at patients currently undergoing oncological treatment as well as survivors who have completed acute treatment. With trajectories and side effects of cancer treatments being rather unpredictable in some cases, patients are expected to miss sessions every now and then. A one-on-one session via telephone, or in case of in-house treatment in person, will be offered to patients to catch up on the content of the missed session. Treatment will be considered completed if patients have attended or caught up with at least eight of the ten sessions in total and have not missed more than four group sessions in total. Pre- and post-intervention sessions are mandatory. Attendance and catch-up-sessions will be registered.

### Outcome measures

#### Feasibility outcomes

Feasibility measures will include recruitment rates and adherence to treatment, suitability of criteria for exclusion and inclusion, suitability of data collection procedures, and suitability of recruitment procedures. Participant flow will be documented according to CONSORT guidelines (38). Challenges in trial organization and changes to amend difficulties will be documented in the study teams meeting protocols. Therapists’ adherence to the intervention manual will be documented using supervision session protocols and the IPT scale of Strategies in Therapy Use Form – Therapist Version (STUF) developed by Swartz et al. (43). Documentation of reasons for drop-out and documentation of the post-intervention session will provide information on previously disregarded patient reported outcomes, suitability of study procedures according to the participants, and unintended effects of the intervention.

Participant adherence to treatment will be used as primary criterion for trial progression, with an adherence rate above 80% indicating progression (‘Green/Go’), an adherence rate between 80% and 40% indicating progression only after thorough revision and amendment (‘Amber/amend’) and an adherence rate below 40% indicating no progression (‘Red/stop’) (44). Status regarding the progression of the trial will be assessed regularly in the study teams meetings and documented in meeting protocols, along with changes to the protocol that might be implemented to amend adherence rates. Qualitative data will be used to inform amendments to study procedure and intervention structure or content.

#### Preliminary efficacy outcomes

Assessment will contain German versions of the following instruments:

- Sociodemographic and medical data (self-generated items)
- Patient Health Questionnaire (PHQ-9) (45–47) (9 items reflecting the diagnostic criteria of DSM-IV depressive disorders)
- General Anxiety Disorder Questionnaire (GAD-7) (48, 49) (7 items reflecting the most prominent diagnostic criteria of DSM-IV generalized anxiety disorder)
- Distress Thermometer analogue scale and problem areas (DT) (50, 51) (screening tool assessing overall distress in cancer patients on a scale of 0 to 10 and listing possible physical and psychosocial problem areas)
- Revised UCLA Loneliness Scale (R-UCLA) (52, 53) (20 items assessing loneliness)
- Brief Social Support Scale (BS6) (54) (6 items assessing perceived emotional and tangible social support)
- Interpersonal Needs Questionnaire (INQ) (55, 56) (15 items assessing the interpersonal concepts of thwarted belongingness and perceived burdensomeness)
- Interpersonal stressors (4 self-generated open-ended questions assessing burdensome relationships, distressing role changes, conflicts)
- Quality of Life (EQ-5D-3L) (57, 58) (5 Items assessing general quality of life in a descriptive system regarding mobility, self-care, usual activities, pain/discomfort and anxiety/depression)
- Hornheider Screening Instrument (HSI) (59) (7 items assessing need for psychosocial care in patients with cancer)

Qualitative data will be used to inform on previously disregarded outcomes and the lived experience of the participants.

The schedule of the assessments is summarized in (**Table 1**). Data will be collected through an online survey designed using RedCap electronic data capture tools hosted at Tübingen University (36, 37). Participants will receive a link to the survey via email. The assessment will take about 30 minutes to complete. It can be paused and accessed again at a later point in time using a personal code. Each measure constitutes one page in the survey. Survey answers cannot be changed once the page has been submitted, and a new page cannot be opened until all items have been answered. Participants will be assigned a random number generated using MATLAB 2023a (60) Random Number Generator with current time as seed (rng(‘shuffle’); rand). All data in the assessment will be recorded under that pseudonym only.

Primary outcome will be symptoms of depression at completion of the treatment and 3-months follow-up, which will be assessed using the Patient Health Questionnaire (PHQ-9). As secondary outcomes we chose symptoms of anxiety, which will be assessed using the Generalized Anxiety Disorder Questionnaire (GAD-7), and psychological distress as measured by the Distress Thermometer (DT) at completion of the treatment and 3-months follow-up. Tertiary measures are self-reports of loneliness as measured by the R-ULCA Loneliness Scale, perceived social support as measured by the Brief Social Support Scale (BS6), interpersonal needs (perceived burdensomeness and thwarted belongingness) as measured by the Interpersonal Needs Questionnaire (INQ) at completion of the treatment and 3-months follow-up.

Qualitative data will consist of the audio recording of the post-intervention semi-structured interviews. Records will be saved using the patient pseudonym on study tablets that are not connected to the internet and will be transcribed by the study team. Recordings will be deleted after analysis is completed. A translation of the interview guidelines can be seen in (**Table 4**).

**Table 4:** Post-intervention semi-structured interview.

Topic	Question
Goal achievement and relevance to everyday life	In the preparatory session, you formulated the following goal for the group therapy: X What has changed in relation to X since the start of therapy? How do you notice the change in everyday life? What would you say - have you achieved, partially achieved or not achieved the goal? Which aspects of the group therapy did you find helpful regarding X? Which aspects did you find inappropriate or not useful? Have there been any changes in your everyday life beyond the goals we just talked about? How do you observe these changes? How is the change related to the group therapy? What interpersonal experiences do you take away from the group?
Challenges and adverse effects	What were the particular challenges or difficulties for you in group therapy? How did the therapists or the group support you in overcoming the challenges? (How could the therapists or the group have supported you better in overcoming the challenges?) Did group participation have adverse effects for you?
Organizational barriers and facilitators	How did you feel about the organizational aspects of group therapy? What were barriers to participation? What facilitated participation? How did you feel about the organizational aspects of the study (consent, information flow, questionnaires, interviews)? What were barriers to participation? What facilitated participation?
Outlook	Looking at the time after the group therapy: What are your goals, what changes do you want to make? How can the therapists or the group support you with that? What are you worried about? How can the therapists or the group support you with that?
Other	Is there anything else you would like to share with us?

### Sample size calculation and statistical analyses

All quantitative data analyses will be conducted using R (61), RStudio (62), and MATLAB (60).

Due to the nested nature of group intervention data (63) and more specifically the scarcity of research regarding IPT in patients with cancer other than breast cancer, sample size calculations are to be treated with caution. A sample size of 40 participants is considered appropriate for this feasibility study.

Feasibility outcomes will be analyzed using percentages. Descriptive analyses of sociodemographic data will be conducted. Preliminary efficacy will be analyzed in within-subject comparisons for each outcome measure and reported with 95% confidence intervals (CI). Results regarding the primary outcome (symptoms of depression, PHQ-9) will be considered indicative of efficacy if the CI is above zero and includes the predefined Minimal Clinically Important Difference (MCID) of 5 points as previously determined by Löwe et al. (64). Results regarding secondary and tertiary outcomes will be considered indicative of efficacy if it is above zero.

Missing data will be reported as such. It is expected that at least a subset of dropouts may nevertheless be available to complete the post-treatment assessment, improving the accuracy of data collected. An interim analysis is not planned.

Qualitative analysis will be conducted using MAXQDA 2022 software (65). Record transcripts will undergo qualitative content analysis as described by Mayring (66) using deductive-inductive category formation, starting with an initial batch of 6 interviews. Iterative analysis will continue until saturation is reached. Procedures and results will be recorded and reported in accordance with the Standards for Reporting Qualitative Research (SRQR) (67). Saturation will be assessed using the 16-item checklist provided by Ahmed (68). Saturation will be defined as lack of emergence of new themes in three consecutive interview transcripts. Meta-analysis indicates a sample size of 13 is enough to reach saturation for most studies (69). Analysis will focus on the following aspects included in the semi-structured interview (**Table 4**): changes in everyday life, helpful elements of group IPT, inappropriate elements of group IPT, interpersonal experiences in group IPT, challenges in group IPT, support regarding challenges, adverse effects, barriers and facilitators for participation in group IPT and study procedures, and outlook.

### Patient and public involvement

Treatment modules were developed by careful revision of themes and struggles most frequently addressed by patients with cancer and their caregivers in individual sessions of psycho-oncology services. They were adapted in an iterative process relying heavily on reports and feedback by participants in three initial trial run-throughs of the treatment.

### Ethics and dissemination

The pilot study has been reviewed and approved by the ethics committee of the faculty of medicine of Eberhard-Karls-University of Tübingen (Reference No 177/2023BO2). The qualitative aspects of the study (audio recording of post-intervention interviews, qualitative content analysis of recordings) were the subject of an amendment to the original review and have not yet been approved. Qualitative data collection and analysis will not commence until approval is received. Results will be published in peer-reviewed journals and conference presentations.

### Trial status

Trial start date: 09/24; currently recruiting (n_current_=6 as of 10/24).

### Registration

The trial was preregistered with Open Source Foundation Registries (Registration DOI: https://doi.org/10.17605/OSF.IO/D4WJ9).

## Discussion

In this pilot study in 40 patients, we propose to develop and evaluate an IPT group treatment for symptoms of depression specifically tailored to the needs of patients with cancer and comorbid symptoms of depression.

The described study is a pilot study, and information gathered will be limited by the exclusive use of self-report data and the naturalistic design without any control group. A lack of resources, potential difficulties in recruiting participants and ethical considerations led to the decision not to apply a randomized controlled design.

The naturalistic group composition of patients with diverse types of cancer and in different treatment stages increases the generalizability of findings from this study. It also adds to its value for care providers within the psycho-oncological services who also treat patients from all specialties and at all points in the patient journey. Participants may even profit from the different perspectives and experiences shared in more heterogenous groups.

Our preliminary findings will suggest whether a trial of group IPT is feasible and indicate preliminary efficacy in reducing symptoms of depression in patients with cancer. We will also assess changes in symptoms of depression, anxiety, and in interpersonal factors such as loneliness, thwarted sense of belonging, perceived burdensomeness, and perceived social support. The findings will inform us how to adapt and change the program in order to make it more efficient or feasible for patients and care providers. Qualitative content analysis will enrich our understanding of the lived experience of participants regarding feasibility and acceptability of intervention and study procedures and provide qualitative insight into everyday changes for patients.

Adequate and efficient treatment for depressive symptoms in patients with cancer is urgently needed. The proposed group IPT program adapted for cancer is a novel, unique and promising approach to the problem. An effective treatment for symptoms of depression in patients with cancer is highly relevant because depressive symptoms affect a large proportion of patients with cancer, and have detrimental effects on patients’ quality of life, treatment compliance, and even mortality (5).

To our knowledge, the treatment is new and unique both in that it provides patients with cancer with group IPT, and in that the group IPT program is tailored to the specific needs of cancer patients. The study is innovative in that it provides the proposed treatment to patients of different cancer types and treatment stages, creating a setting that is naturalistic and realistic in the context of psycho-oncology services.

## Data Availability

The original contributions presented in the study are included in the article/supplementary material. Further inquiries can be directed to the corresponding author.
